# Prospective Safety Surveillance of GH-Deficient Adults: Comparison of GH-Treated vs Untreated Patients

**DOI:** 10.1210/jc.2012-2684

**Published:** 2013-01-23

**Authors:** Mark L. Hartman, Rong Xu, Brenda J. Crowe, Leslie L. Robison, Eva Marie Erfurth, David L. Kleinberg, Alan G. Zimmermann, Whitney W. Woodmansee, Gordon B. Cutler, John J. Chipman, Shlomo Melmed

**Affiliations:** Lilly Research Laboratories (M.L.H., R.X., B.J.C., A.G.Z., G.B.C., J.J.C.), Indianapolis, Indiana 46285; St. Jude Children's Research Hospital (L.L.R.), Memphis, Tennessee 38105; Skånes University Hospital (E.M.E.), SE-221 85 Lund, Sweden; New York University Medical Center (D.L.K.), New York, New York 10016; Brigham and Women's Hospital (W.W.W.), Harvard Medical School, Boston, Massachusetts 02115; and Cedars-Sinai Medical Center (S.M.), University of California at Los Angeles School of Medicine, Los Angeles, California 90048

## Abstract

**Context:**

In clinical practice, the safety profile of GH replacement therapy for GH-deficient adults compared with no replacement therapy is unknown.

**Objective:**

The objective of this study was to compare adverse events (AEs) in GH-deficient adults who were GH-treated with those in GH-deficient adults who did not receive GH replacement.

**Design and Setting:**

This was a prospective observational study in the setting of US clinical practices.

**Patients and Outcome Measures:**

AEs were compared between GH-treated (n = 1988) and untreated (n = 442) GH-deficient adults after adjusting for baseline group differences and controlling the false discovery rate. The standardized mortality ratio was calculated using US mortality rates.

**Results:**

After a mean follow-up of 2.3 years, there was no significant difference in rates of death, cancer, intracranial tumor growth or recurrence, diabetes, or cardiovascular events in GH-treated compared with untreated patients. The standardized mortality ratio was not increased in either group. Unexpected AEs (GH-treated vs untreated, *P* ≤ .05) included insomnia (6.4% vs 2.7%), dyspnea (4.2% vs 2.0%), anxiety (3.4% vs 0.9%), sleep apnea (3.3% vs 0.9%), and decreased libido (2.1% vs 0.2%). Some of these AEs were related to baseline risk factors (including obesity and cardiopulmonary disease), higher GH dose, or concomitant GH side effects.

**Conclusions:**

In GH-deficient adults, there was no evidence for a GH treatment effect on death, cancer, intracranial tumor recurrence, diabetes, or cardiovascular events, although the follow-up period was of insufficient duration to be conclusive for these long-term events. The identification of unexpected GH-related AEs reinforces the fact that patient selection and GH dose titration are important to ensure safety of adult GH replacement.

GH is approved in various countries for treatment of adult GH deficiency (GHD) due to hypothalamic or pituitary disease, pediatric short stature due to several causes, and certain catabolic states (HIV-associated muscle wasting and short bowel syndrome). The increasing use of GH for these indications, for unapproved indications, and for sports doping (1) underscores the importance of safety information from rigorous large-scale studies. This information is especially important for adults with GHD who may receive lifelong GH replacement.

Regulatory approval of GH treatment for adult GHD was based on placebo-controlled clinical trials of 6 to 12 months' duration, each with <200 patients (2). Because uncommon adverse drug reactions cannot be detected reliably in studies of this size, postmarketing research programs with larger sample sizes have been conducted to expand the safety data for adult GH replacement (3–6). Although such studies have been generally reassuring, they have not compared the outcomes of GH-treated and GH-untreated patients in a prospective observational cohort. Such comparison is needed because hypopituitarism itself may increase rates of myocardial infarction, cerebrovascular events, malignancies, and overall mortality (7). Scientific societies have also recommended additional surveillance for diabetes, tumor recurrence, de novo tumors, and potential unforeseen adverse effects (8).

Unlike GH surveillance programs that lack a control group (9), the US Hypopituitary Control and Complication Study (HypoCCS) was designed to compare the incidence rate of events between GH-treated and untreated GH-deficient adult patients who had similar hypothalamic-pituitary disorders over a prospective follow-up period of 5 years. Here, we report the safety profile of GH treatment in patients with adult GHD compared with similar patients not receiving GH treatment. The mortality rates for both treatment groups were compared with that of the US general population.

## Subjects and Methods

### Study design

The US HypoCCS was a prospective observational study sponsored by Eli Lilly and Company to examine long-term safety of GH (Humatrope; Eli Lilly and Company, Indianapolis, Indiana) treatment in adults with GHD. Investigators at 157 US centers participated between 1996 and 2002 (10). Data were verified against source documents by monitors reviewing patient records at the sites (data source verification). The 2430 subjects (1988 GH-treated and 442 untreated) who enrolled in US HypoCCS and had follow-up data comprise the focus of this report.

In 2002, a new global HypoCCS study was launched, merging the US and European HypoCCS studies, with some differences from the US study in data collection; most notably, data source verification was not performed. Patients who participated in US HypoCCS were allowed to enroll in the new global HypoCCS, although only a subset were subsequently enrolled. To evaluate a selected number of new safety signals identified during analysis of US HypoCCS, an interim analysis of the US patients enrolled in the global study was performed in 2008, with a focus on new patients who had not previously participated in US HypoCCS (1034 GH-treated and 233 untreated). Local institutional review boards approved both protocols; patients provided written informed consent.

The study design, inclusion/exclusion criteria, and hormone assay methods for US HypoCCS have been described previously (10, 11). Enrolled patients met the criteria for adult GHD as specified in the US package insert for Humatrope (10, 11). According to the observational study design, the choice of whether to receive GH replacement therapy or remain untreated was made by each patient in consultation with his or her endocrinologist. Investigators were allowed to individualize GH treatment based on the clinical and biochemical (serum IGF-I and IGF binding protein-3 [IGFBP-3]) responses of each patient; the protocol recommended a starting dose of not more than 6 μg/kg/d and a maximum dose of 12.5 μg/kg/d based on the US package insert for Humatrope as worded at the beginning of the study. Follow-up visits were at 6-month intervals (±1 month) for both treatment groups. All patients who met the diagnostic criteria and had at least 1 follow-up visit were analyzed.

### Adverse event (AE) reporting

A treatment-emergent adverse event (TEAE) was defined as a condition that developed or was present but worsened in severity after enrollment in the study. Serious adverse events (SAEs) were reported as defined by regulatory criteria (life-threatening, hospitalization, severe disability, congenital anomaly, cancer, drug overdose, death, or investigator-designated as serious for other reason). To ensure accuracy of event descriptions, investigators were contacted for follow-up information on all SAEs. AEs were analyzed based on individual preferred terms defined by the Medical Dictionary for Regulatory Activities (MedDRA, version 7.0), as recommended by the International Conference for Harmonization and adopted by most regulatory authorities worldwide. TEAEs were classified as expected if they were previously reported side effects of GH treatment (2, 12).

### Baseline patient characteristics

Baseline patient characteristics are summarized in Table 1. Untreated patients were older, had more preexisting medical problems, and were more likely to be male and to have an intracranial tumor (including pituitary adenoma) as the cause of GHD. The proportions of patients with specific pituitary hormone deficiencies and hormonal replacements were similar, except that diabetes insipidus and estrogen replacement (among women) were more commonly encountered in GH-treated patients.

**Table 1.** Patient Characteristics

Characteristic	GH-Treated (n = 1988^a^)	Untreated (n = 442^a^)	*P* Value^b^
Follow-up, y	2.3 ± 1.4	2.3 ± 1.6	.555
Age at entry, y	46 ± 15	55 ± 16	<.001
Sex (male/female), %	56/44	62/38	.023
Body mass index, kg/m^2^	31 ± 7	30 ± 6	.002
History of smoking, y	7 ± 12	9 ± 14	.006
Intracranial tumor as cause of GHD, %	63	76	<.001
Radiotherapy associated with GHD, %	29	32	.257
Isolated GH deficiency, %	13	10	.032
Onset of GHD (adult/childhood), %	84/16	88/12	.023
GH therapy before study entry, %			
Adult-onset GHD	12	5	<.001
Childhood-onset GHD	72	59	.051
Preexisting medical problem, %			
Visual impairment	27	34	.002
Hypertension	25	33	.001
Hyperlipidemia	42	50	.003
Diabetes mellitus	8	15	<.001
Coronary artery disease	6	12	<.001
Pituitary hormone deficiency, %^c^			
Secondary hypothyroidism	72	75	.163
Thyroid hormone replacement	97	95	.013
Secondary hypoadrenalism	52	56	.083
Glucocorticoid replacement	95	95	.783
Secondary hypogonadism (men)	82	85	.302
Androgen replacement	88	84	.117
Secondary hypogonadism (women)	52	51	.783
Estrogen replacement	64	48	.004
Diabetes insipidus	22	13	<.001
Vasopressin replacement	88	84	.439
Serum IGF-I, μg/L	108 ± 61	90 ± 51	<.001
Serum IGF-I SD score	−2.6 ± 1.9	−2.5 ± 1.7	.695
Serum IGFBP-3, μg/L	2.4 ± 0.9	2.1 ± 1.0	<.001

a The number of subjects is smaller for some variables.

b Comparisons unadjusted for propensity score. χ^2^ test for categorical variables; *t* test for continuous variables (mean ± SD shown).

c Percentage of patients with specific pituitary hormone deficiencies and of those the percentage receiving hormonal replacement.

### Statistical analysis

To adjust for imbalances in patient characteristics between GH-treated and untreated groups due to the observational study design, stratified propensity score analysis was used for group comparisons of TEAEs and SAEs (13). Propensity scores (conditional probability of being treated) were derived from a logistic regression model that included 37 covariates, selected because of a baseline imbalance between treatment groups or their perceived impact on the occurrence of AEs. (Details of the 37 covariates used in the logistic regression model are found in Supplemental Methods published on The Endocrine Society's Journals Online web site at http://jcem.endojournals.org.) Missing quantitative baseline data were imputed by treatment mean imputation (14). Patients were stratified into quintiles based on the propensity scores (15). Consequently, 97% of baseline covariate comparisons within propensity score quintiles showed no statistically significant treatment group differences. TEAE and SAE rates were compared using the Cochran-Mantel-Haenszel test, controlling for the propensity score quintiles (thus controlling for baseline differences). A *P* value of ≤.05 was considered significant.

Because hundreds of treatment group comparisons were performed, multiplicity adjustment was necessary. The false discovery rate (FDR) method was used to distinguish between probable false positives and probable true findings from the propensity score analysis (16). Different from traditional approaches to multiplicity adjustment (eg, the Bonferroni procedure), which control the probability of making 1 or more false discoveries (type I error), the FDR controls the expected proportion of false positives while maintaining power to uncover real differences. We eliminated from the analysis TEAEs with incidence rates so low (<0.5% in both groups) that achieving a statistically significant treatment difference was unlikely (16). Within each MedDRA system organ class, a *P* value adjusted to control the FDR was calculated for each of the remaining TEAEs and compared with the prespecified cutoff of 10% (16). For this analysis, a 10% false-positive rate among the significant findings was considered acceptable. We report all TEAEs with an incidence of >1.5% in either treatment group that were significantly more common (*P* ≤ .05) in GH-treated than in untreated patients and provide the FDR-adjusted *P* value for interpretation of these events as “probable true positives” or “probable false positives.” Associations between unexpected TEAEs and other potentially related conditions were examined via logistic regression of the unexpected event on the condition of interest, adjusting for therapy and propensity score quintile.

Standardized mortality ratios (SMRs) were used to quantify the risk of death among the GH-treated and untreated groups. To compute the SMR, an expected number of deaths was calculated using age- and sex-specific US mortality rates, reported by the National Center for Health Statistics. Person-years of observation for the patient population were applied to age-specific rates for 5-year age intervals to calculate all-cause SMRs and the corresponding 95% confidence interval (CI) (17).

## Results

### Follow-up time in study

Mean follow-up time did not differ between GH-treated and untreated patients (Table 1). Median (25th percentile, 75th percentile, maximum) follow-up was 2.2 (1.1, 3.5, 5.5) years for GH-treated patients and 2.1 (1.0, 3.8, 5.2) years for untreated patients.

### Serum IGF-I and IGFBP-3 concentrations

Mean baseline serum concentrations of IGF-I and IGFBP-3 are shown in Table 1; serum IGF-I standard deviation scores (based on age-adjusted normative data, measured in a central laboratory) were <−2 in 56% and 54% of GH-treated and untreated patients, respectively (*P* = .406). At follow-up visits, the percentage of GH-treated patients with elevated (SD score ≥2) and low (SD score <−2) IGF-I concentrations ranged from 0% to 2.0% and 4.8% to 18.5%, respectively.

### TEAEs

Overall, the incidence of TEAEs was higher in GH-treated than in untreated patients (84.1% vs 69.2%, *P* < .001). Table 2 lists TEAEs that were more common (*P* ≤ .05) in GH-treated than in untreated patients, after adjusting for baseline differences. After application of the FDR criteria (*P* ≤ .05 and FDR-adjusted *P* ≤ .1), most expected TEAEs were retained as probable true positives, with the exception of joint swelling, joint stiffness, and acne. Conversely, several unexpected events were identified as probable false positives.

**Table 2.** TEAEs That Were Significantly More Common (*P* ≤ .05) in the GH-Treated Group Than in the Untreated Group After Adjusting for Baseline Differences^a^

AEs	GH-Treated (n = 1988)	Untreated (n = 442)	*P* Value^b^	FDR-Adjusted *P* Value
Expected events				
Probable true positive, n (%)				
Arthralgia	397 (20.0)	32 (7.2)	<.001	<.001
Edema peripheral	307 (15.4)	26 (5.9)	<.001	<.001
Back pain	175 (8.8)	18 (4.1)	.004	.056
Hypoesthesia	103 (5.2)	10 (2.3)	.009	.098
Myalgia	100 (5.0)	9 (2.0)	.006	.059
Energy increased	90 (4.5)	0 (0.0)	<.001	<.001
Paresthesia	85 (4.3)	7 (1.6)	.009	.098
Carpal tunnel syndrome	79 (4.0)	5 (1.1)	.014	.103
Hormone level abnormal^c^	43 (2.2)	1 (0.2)	.003	.037
IGF-I increased^d^	37 (1.9)	1 (0.2)	.008	.055
Probable false positive, n (%)				
Joint swelling	51 (2.6)	5 (1.1)	.037	.213
Acne	40 (2.0)	1 (0.2)	.046	.228
Joint stiffness	35 (1.8)	1 (0.2)	.021	.154
Unexpected events				
Probable true positive, n (%)				
Insomnia	128 (6.4)	12 (2.7)	.014	.090
Dyspnea	83 (4.2)	9 (2.0)	.006	.090
Anxiety	67 (3.4)	4 (0.9)	.021	.090
Sleep apnea syndrome	66 (3.3)	4 (0.9)	.010	.098
Libido decreased	42 (2.1)	1 (0.2)	.016	.090
Probable false positive, n (%)				
Headache	276 (13.9)	43 (9.7)	.038	.182
Depression	195 (9.8)	23 (5.2)	.041	.127
Nausea	163 (8.2)	24 (5.4)	.040	.417
Hypertension	144 (7.2)	23 (5.2)	.050	.132
Nasopharyngitis	92 (4.6)	11 (2.5)	.042	.503
Abdominal distension	33 (1.7)	2 (0.5)	.045	.417
Asthma	33 (1.7)	1 (0.2)	.044	.350

a Events (MedDRA preferred terms) were defined as “expected” or “unexpected” based on previous studies. An event was considered a “probable true positive” if FDR-adjusted *P* ≤ .1. AEs with an incidence >0.5% in either group were included in the statistical analysis; events with an incidence of >1.5% are shown in the table.

b Cochran-Mantel-Haenszel general association.

c Includes abnormal serum concentrations of IGF-I, IGFBP-3, and dehydroepiandrosterone.

d Indicates an increase in serum IGF-I that the investigator considered to be an AE.

The incidence of TEAEs related to benign or malignant neoplasms did not differ between groups (GH-treated, 8.1%; untreated, 10.0%; *P* = .77). For MedDRA preferred terms related to glucose metabolism and diabetes mellitus, frequencies of TEAEs were <2%, and there were no significant group differences. Cardiac or vascular disorders were not identified as probable true-positive findings.

### Unexpected events in GH-treated patients

Five TEAEs in the GH-treated group were identified as probable true positives but were unexpected based on previous studies (Table 2). For GH-treated patients, reported events of insomnia, dyspnea, anxiety, sleep apnea, and decreased libido were mild to moderate in severity (as reported by the investigator) in 93%, 92%, 82%, 85%, and 90% of patients, respectively, and were reported during the first year of GH treatment in 58%, 61%, 52%, 44%, and 55% of patients, respectively.

To further clarify the nature of these unexpected TEAEs, possible clinical associations were examined. Sleep apnea was associated with both obesity (73% had a body mass index ≥30 kg/m^2^; *P* = .003) and fluid retention (*P* < .001). Dyspnea was associated with fluid retention (*P* < .001). Insomnia was associated with anxiety (*P* < .001); both insomnia and anxiety were associated with painful AEs (such as musculoskeletal disorders and others) (*P* < .001). Decreased libido was not associated with anxiety, depression, or painful AEs. Among patients reporting dyspnea, 78% had a related disorder or symptom (cardiac or respiratory disorder, infection, or edema). Among those reporting anxiety, 33% either had anxiety listed as a preexisting condition or were taking anxiolytic medication at baseline. For those with decreased libido, most were hypogonadal and/or menopausal at baseline (87% women and 85% men); a minority of these hypogonadal patients were not receiving hormonal replacement when decreased libido was reported (15% women and 26% men).

Compared with the overall study population followed for 2 years, GH-treated patients reporting sleep apnea more commonly had higher (≥0.8 mg/d) GH doses (26% vs 14% for all GH-treated patients) and elevated serum IGF-I concentrations (35% vs 14% for all GH-treated patients), but this association was not observed for the other 4 unexpected events. During follow-up, reduction in clinical severity was reported in 28%, 49%, 25%, 18%, and 19% of GH-treated patients experiencing insomnia, dyspnea, anxiety, sleep apnea, and decreased libido, respectively. Of these, a reduction of the GH dose was associated with clinical severity decrease in 25%, 17%, 35%, 33%, and 50% of those subjects with insomnia, dyspnea, anxiety, sleep apnea, and decreased libido, respectively.

To evaluate these unexpected findings further, an independent interim analysis of US patients subsequently enrolled in the global HypoCCS was performed in 2008 (mean follow-up period, 2.0 years; range, 0.1–5.4 years). This analysis examined (using the same methodology) 4 events of particular interest (dyspnea, sleep apnea, hypertension, and decreased libido) based on the findings reported herein, reviews of published literature, and the Lilly spontaneous AE database. Among US patients who had not previously participated in US HypoCCS (1034 GH-treated and 233 untreated), there were no statistically significant differences in the proportion of GH-treated and untreated patients reporting dyspnea (1.5% vs 0.9%, respectively), sleep apnea (1.6% vs 2.6%), hypertension (3.8% vs 2.1%), or decreased libido (0.2% vs 0%).

### SAEs

After controlling for baseline group differences, the proportion of GH-treated and untreated patients experiencing death, cancer, or benign extracranial tumors or cysts (de novo or recurrent) did not differ (Tables 3, 4, and 5). Among patients with a previous intracranial tumor and evaluable baseline data, no difference in growth or recurrence rates of pituitary adenoma, craniopharyngioma, or other intracranial tumors was observed between GH-treated and untreated patients (Table 6).

**Table 3.** Summary of Number of SAEs Reported as Deaths^a^

	GH-Treated (n = 1988)	Untreated (n = 442)
Cause of death, n (%)		
Cause unknown or not specified	9 (0.45)	3 (0.68)
Cardiac arrhythmia or arrest	6 (0.30)	1 (0.23)
Sepsis, pneumonia, or other infection	3 (0.15)	1 (0.23)
Myocardial infarction	2 (0.10)	0
Cerebrovascular accident or cerebral hemorrhage	1 (0.05)	2 (0.45)
Suicide	3 (0.15)	0
Respiratory failure or arrest	3 (0.15)	0
Cardiac failure	1 (0.05)	2 (0.45)
Motor vehicle accident	1 (0.05)	2 (0.45)
Acute myeloid leukemia	1 (0.05)	0
Astrocytoma (malignant)	1 (0.05)	0
Ruptured aortic aneurysm	1 (0.05)	0
Carbon monoxide poisoning	1 (0.05)	0
Total deaths, n (%)	33 (1.66)	11 (2.49)

a There was no significant difference in the total proportions of patients dying in the 2 groups after controlling for baseline group differences (*P* = .73).

**Table 4.** Summary of Number of SAEs Related to Cancer (Either New Cancers or Recurrence of Previous Disease)^a^

	GH-Treated (n = 1988)	Untreated (n = 442)
Cancer type, n (%)		
Skin cancer^b^	10 (0.50)	5 (1.13)
Prostate cancer	3 (0.15)	3 (0.68)
Breast cancer	4 (0.20)	1 (0.23)
Lung cancer^c^	4 (0.20)	0
Colorectal cancer	2 (0.10)	1 (0.23)
Acute leukemia	2 (0.10)	0
Carcinoid tumor	0	1 (0.23)
Lymphoma	0	1 (0.23)
Ovarian cancer	1 (0.05)	0
Ewing's sarcoma	1 (0.05)	0
Pancreatic islet cell tumor	1 (0.05)	0
Bladder/urethral cancer	1 (0.05)	0
Fibrosarcoma	1 (0.05)	0
Laryngeal cancer	1 (0.05)	0
Polycythemia vera	1 (0.05)	0
Total cancers, n (%)	32 (1.61)	12 (2.71)

a For total cancer events, there was no significant difference between the 2 groups after controlling for baseline differences (*P* = .57).

b Skin cancers comprised 10 basal cell carcinomas (6 GH-treated patients and 4 untreated patients), 3 melanomas (3 GH-treated patients), and 2 squamous cell carcinomas (1 GH-treated patient and 1 untreated patient); 1 of the patients with a melanoma also had a basal cell carcinoma that is not included in the table.

c Includes 1 patient with a pulmonary mass that was presumed to be a lung cancer by the oncologist, but a tissue diagnosis was not obtained.

**Table 5.** Summary of Number of SAEs Related to Benign Extracranial Tumors or Cysts (Either New Tumors or Progression of Preexisting Tumors)^a^

	GH-Treated (n = 1988)	Untreated (n = 442)
Tumor type, n (%)		
Uterine leiomyoma	3 (0.15)	0
Ovarian cyst or adenoma	3 (0.15)	0
Hemangioma	2 (0.10)	0
Histiocytosis	0	1 (0.23)
Colon adenoma	1 (0.05)	0
Lipoma	1 (0.05)	0
Synovial cyst	1 (0.05)	0
Total benign tumors and cysts, n (%)	11 (0.55)	1 (0.23)

a For total events, there was no significant difference between the 2 groups after controlling for baseline group differences (*P* = .46).

**Table 6.** SAEs Related to Growth or Recurrence of Preexisting Intracranial Tumors Including Pituitary Adenomas^a^

Intracranial Tumor	GH-Treated	Untreated	*P* Value
Pituitary adenomas	24/965 (2.5%)	12/273 (4.4%)	.21
Craniopharyngiomas	8/211 (3.8%)	2/36 (5.6%)	.82
Other intracranial tumors^b^	4/133 (3.0%)	1/30 (3.3%)	.90

a For each category, the denominator is patients with a history of the tumor before study entry. *P* values have been adjusted for baseline group differences.

b The other intracranial tumor events included 2 meningiomas (both GH-treated patients), 1 astrocytoma (GH-treated patient), 1 medulloblastoma (GH-treated patient), and 1 Rathke cleft cyst (untreated patient).

### Comparison with US general population

The all-cause SMR was not increased in either treatment group (GH-treated, 0.86 [95% CI, 0.59–1.21]; untreated, 0.58 [95% CI, 0.29–1.04]) and did not differ significantly between GH-treated and untreated patients after 4655 and 1019 person-years of observation, respectively (*P* = .26).

## Discussion

We compared the safety profile of GH treatment vs no treatment in patients with adult GHD in the setting of routine clinical practice and identified unexpected AEs not previously associated with GH treatment. Notably, sleep apnea and dyspnea were identified as new risks of GH treatment. Although the mean follow-up period (2.3 years) was of insufficient duration to be conclusive, there was no increased rate of death, new cancer, intracranial tumor recurrence, diabetes mellitus, or cardiovascular events in GH-treated patients compared with untreated patients. In addition, the SMR compared with that of the general US population was not increased in either group.

A prerequisite to these findings was use of an analytical approach that would reduce biases inherent in the observational study design. Untreated patients were older, sicker, and more likely to have an intracranial tumor as the cause of GHD than GH-treated patients. Thus, the decision whether to replace GH was influenced by medical history. This selection bias was reduced by stratifying analyses within patient subgroups, balanced for baseline covariates, using the propensity score method (13, 15). In addition, we used a statistical method to control the proportion of false-positive findings while maintaining the ability to detect new safety concerns (16). These methods provided robust analysis of observational data.

Because an association has been reported between serum IGF-I levels and prostate, breast, and colon cancer risk, increases in GH/IGF-I levels during medically indicated GH replacement have been considered a potential safety concern (18, 19). Although specific cancers in the current study were too few for analysis by tumor type, no concerning trends were noted, considering the greater than 4-fold difference in treatment group size. Similarly, the absence of a GH effect on overall de novo cancer occurrence and intracranial tumor recurrence is reassuring and consistent with previous reports in adults with GHD (3, 4, 6, 20, 21), with the caveat that longer-term follow-up is needed. In addition, a recent analysis of global HypoCCS data revealed a standardized incidence ratio for all cancers of 0.88 (95% CI, 0.74–1.04) in GH-treated patients globally and, among US patients, the standardized incidence ratio was 0.94 (95% CI, 0.73–1.18) for GH-treated patients and 1.16 (95% CI, 0.76–1.69) for untreated patients (6). In long-term follow-up studies of adults previously treated during childhood with human pituitary GH, the risk of dying from cancer (specifically, colorectal cancer and Hodgkin disease) was increased in 1 UK study of 1848 patients (22) but not in a US study of 6107 patients (23). All-type cancer-related mortality was not increased in recent reports from the Safety and Appropriateness of Growth Hormone Treatments in Europe study (24, 25). Childhood cancer survivors subsequently treated with GH had an increased risk of a second neoplasm but not of recurrence of the first neoplasm (26, 27). Patients with childhood-onset GHD represented a small minority of subjects enrolled in US HypoCCS, so they were not analyzed separately.

Most AEs identified as GH-related in the current study were expected based on previous short-term controlled trials (2, 12), supporting the validity of the current statistical approach. Although GH replacement in adults with GHD may result in increased fasting glucose concentrations (10), the incidence of diabetes mellitus was not significantly increased, in agreement with a recent report (5). However, 5 unexpected AEs were encountered as significantly more frequent in GH-treated than in untreated patients: sleep apnea, dyspnea, insomnia, anxiety, and decreased libido (occurrence rates, 2%–6%). These events often occurred in patients with predisposing factors, such as high body mass index (sleep apnea) or cardiopulmonary disease (dyspnea), or in whom the condition was either preexisting and already treated (anxiety) or chronologically related to known AEs likely to explain the symptoms (sleep apnea and dyspnea associated with fluid retention or insomnia associated with musculoskeletal pain). Events were observed commonly during the first year of GH treatment, and a substantial proportion decreased in severity after GH dose reduction. Although our analysis sought to constrain the false-positive rate to 10%, it is possible that 1 or 2 of these 5 events may be a false-positive finding.

The increased incidences of sleep apnea and dyspnea in GH-treated than in untreated patients are important unexpected findings. Our results suggest that sleep apnea may be unmasked or precipitated by GH replacement in patients already at risk for sleep apnea (eg, obese patients), particularly those receiving higher GH doses and those exhibiting higher serum IGF-I levels. This finding is consistent with observations in untreated acromegaly (28) and in GH-treated patients with Prader-Willi syndrome (29). Results of polysomnographic studies in adults with GHD have been conflicting. One series of 5 cases described improvement in sleep apnea after discontinuation of GH (30). However, a prospective, uncontrolled study using lower doses of GH reported a high prevalence of sleep apnea in untreated adults with GHD (12 of 19 patients) but no induction or aggravation of sleep apnea with GH treatment (31). Our results also suggest that GH therapy, which commonly causes dose-related edema, may increase the risk of dyspnea in patients with cardiac and respiratory disorders. These findings emphasize the importance of careful GH dose titration to achieve IGF-I levels within the age-adjusted normal range and to avoid edema (8), particularly in patients with preexisting obesity or cardiopulmonary disorders.

After US HypoCCS closed in 2002, individualized GH dosing, which decreases the occurrence of edema, has become more common in clinical practice (8). Our 2008 interim analysis of US patients enrolled in global HypoCCS did not identify a significant difference in reported rates for dyspnea and sleep apnea between GH-treated and untreated patients. Factors other than individualized dosing that may explain this difference include the smaller number of patients in the interim analysis (1267 vs 2430), differences in study design (no source data verification in global HypoCCS), and changes in patient selection over time. The diagnostic characteristics of GH-deficient patients included in the 3 HypoCCS protocols over the period 1996–2005 have changed significantly over this decade; for example, the proportion of patients harboring pituitary adenomas before entry has decreased from 50.2% to 38.6% (32).

Mild to moderate anxiety and insomnia were associated with known GH AEs such as musculoskeletal pain. Anxiety associated with initiation of GH replacement may also result in insomnia. The finding of decreased libido may reflect reporting bias; it was not associated with anxiety, depression, or painful AEs, and only a minority of patients had untreated hypogonadism. In addition, in the 2008 interim analysis of US patients who enrolled in the global HypoCCS after 2002, only 0.2% of GH-treated patients reported decreased libido. Quality-of-life data were not collected in the current study, but results from the European arm of HypoCCS showed improved quality of life during GH replacement in routine clinical practice (33). In a separate study, the ability to become sexually aroused was significantly decreased at baseline in GH-deficient adults compared with age- and sex-matched control subjects and improved after 6 months of GH treatment (34).

This observational study has several inherent limitations. Importantly, the mean follow-up period is short in relation to the longer latency period for cancers to appear. Thus, these results cannot exclude the possibility that longer-term GH treatment might be associated with a higher risk for cancer. Nonetheless, our study was of sufficient size and duration to identify TEAEs not previously identified. The cause of death was not ascertained for 12 of 44 reported deaths, despite investigator efforts to obtain these data from the primary care physicians. The propensity score methodology cannot control for unmeasured baseline imbalances, and it is not known whether there were socioeconomic differences between GH-treated and untreated patients. Because the study was not blinded, reporting bias probably occurred, consisting potentially of closer monitoring of GH-treated patients by physicians and increased reporting of unrelated AEs by patients. The latter, termed the *nocebo phenomenon*, occurs commonly when patients starting new medications have had AEs with other drugs or have preexisting symptoms that predispose them to attribute new or worsening events to the medication (35). No statistical methods are available to control for reporting bias.

In summary, this observational US study of GH-treated and GH-untreated patients with adult GHD demonstrates a safety profile for adult GH replacement therapy similar to that observed in clinical trials. At a mean follow-up of 2.3 years, there is no evidence for an effect of GH therapy on deaths, cancers, diabetes mellitus, cardiovascular events, and intracranial tumor growth or recurrence. Careful GH dose titration is recommended for patients who may be at risk for sleep apnea or cardiopulmonary disorders. As treatment paradigms evolve and long-term surveillance continues, changes in the safety profile of GH replacement therapy may be expected. Notably, GH side effects reported here should not be extrapolated to nonapproved GH uses in pituitary-replete adults (1).
